# Fatiguing exercise reduces cellular passive Young's modulus in human vastus lateralis muscle

**DOI:** 10.1113/EP092072

**Published:** 2024-08-20

**Authors:** Grace E. Privett, Austin W. Ricci, Larry L. David, Karen Wiedenfeld Needham, Yong How Tan, Karina H. Nakayama, Damien M. Callahan

**Affiliations:** ^1^ Department of Human Physiology University of Oregon Eugene Oregon USA; ^2^ Department of Integrative Biosciences, School of Dentistry Oregon Health and Science University Portland Oregon USA; ^3^ Department of Biomedical Engineering Oregon Health and Science University Portland Oregon USA

**Keywords:** cellular stiffness, fatigue, passive mechanics, skeletal muscle, titin

## Abstract

**Abstract:**

Previous studies demonstrated that acute fatiguing exercise transiently reduces whole‐muscle stiffness, which might contribute to increased risk of injury and impaired contractile performance. We sought to elucidate potential intracellular mechanisms underlying these reductions. To that end, the cellular passive Young's modulus was measured in muscle fibres from healthy, young males and females. Eight volunteers (four male and four female) completed unilateral, repeated maximal voluntary knee extensions until task failure, immediately followed by bilateral percutaneous needle muscle biopsy of the post‐fatigued followed by the non‐fatigued control vastus lateralis. Muscle samples were processed for mechanical assessment and separately for imaging and phosphoproteomics. Fibres were passively (pCa 8.0) stretched incrementally to 156% of initial sarcomere length to assess Young's modulus, calculated as the slope of the resulting stress–strain curve at short (sarcomere length = 2.4–3.0 µm) and long (sarcomere length = 3.2–3.8 µm) lengths. Titin phosphorylation was assessed by liquid chromatography followed by high‐resolution mass spectrometry. The passive modulus was significantly reduced in post‐fatigued versus control fibres from male, but not female, participants. Post‐fatigued samples showed altered phosphorylation of five serine residues (four located within the elastic region of titin) but did not exhibit altered active tension or sarcomere ultrastructure. Collectively, these results suggest that acute fatigue is sufficient to alter phosphorylation of skeletal titin in multiple locations. We also found reductions in the passive modulus, consistent with prior reports in the literature investigating striated muscle stiffness. These results provide mechanistic insight contributing to the understanding of dynamic regulation of whole‐muscle tissue mechanics in vivo.

**Highlights:**

- **What is the central question of this study?**
  Previous studies have shown that skeletal muscle stiffness is reduced following a single bout of fatiguing exercise in whole muscle, but it is not known whether these changes manifest at the cellular level, and their potential mechanisms remain unexplored.
- **What is the main finding and its importance?**
  Fatiguing exercise reduces cellular stiffness in skeletal muscle from males but not females, suggesting that fatigue alters tissue compliance in a sex‐dependent manner. The phosphorylation status of titin, a potential mediator of skeletal muscle cellular stiffness, is modified by fatiguing exercise.
- Previous studies have shown that passive skeletal muscle stiffness is reduced following a single bout of fatiguing exercise.
- Lower muscle passive stiffness following fatiguing exercise might increase risk for soft‐tissue injury; however, the underlying mechanisms of this change are unclear.
- Our findings show that fatiguing exercise reduces the passive Young's modulus in skeletal muscle cells from males but not females, suggesting that intracellular proteins contribute to reduced muscle stiffness following repeated loading to task failure in a sex‐dependent manner.
- The phosphorylation status of the intracellular protein titin is modified by fatiguing exercise in a way that might contribute to altered muscle stiffness after fatiguing exercise.
- These results provide important mechanistic insight that might help to explain why biological sex impacts the risk for soft‐tissue injury with repeated or high‐intensity mechanical loading in athletes and the risk of falls in older adults.

## 1 INTRODUCTION

Skeletal muscle stiffness is a crucial characteristic of muscle function. By influencing the rate and efficiency of force transduction from the muscle to the tendon (Wilson & Flanagan, 2008), joint range of motion (Wilson et al., 1991) and joint stability (Blackburn et al., 2011), muscle stiffness impacts locomotion and functionality in everyday tasks. Previous studies using shear‐wave elastography (Andonian et al., 2016; Chalchat et al., 2020; Siracusa et al., 2019) and B‐mode ultrasound (Kubo & Ikebukuro, 2019) suggest that fatiguing exercise reduces whole skeletal muscle stiffness in passive and active conditions. Given that fatigue also affects joint kinematics (Kernozek et al., 2008) and neuromuscular recruitment (Bouillard et al., 2014; De Ste Croix et al., 2015), a fatigue‐induced reduction of skeletal muscle stiffness might contribute to knee joint destabilization and subsequent soft‐tissue injury in athletes (Blackburn et al., 2011; Myer et al., 2008a; Watsford et al., 2010). Specifically, reduced muscle stiffness decreases the amount of strain elastic energy that can be absorbed by a tissue before injury occurs (Mair et al., 1996). When experienced in older adults, fatigue‐induced muscle compliance can reduce joint stability and impair the ability to adjust muscle function instantly in response to perturbation, collectively contributing to an increased risk of falls following repeated and/or prolonged muscle activation (Morrison et al., 2016). Despite the clear clinical implications of reduced musculotendinous stiffness after fatiguing exercise, the underlying mechanisms of this phenomenon and whether they are mediated by biological sex are not yet known.

Previous studies of fatigue‐induced reductions of whole‐muscle stiffness included exclusively (Chalchat et al., 2020; Siracusa et al., 2019) or predominantly (Andonian et al., 2016) male participants. However, musculotendinous stiffness appears to be influenced by biological sex, with women exhibiting reduced passive stiffness of the gastrocnemius (Morse, 2011) and reduced active stiffness of the medial gastrocnemius tendon (Kubo et al., 2003) in comparison to men. These divergent mechanical properties have been attributed to the effects of sex hormones on whole‐muscle (Chidi‐Ogbolu & Baar, 2019; Ham et al., 2020) and tendon (Hansen & Kjaer, 2016) stiffness. In muscle, oestrogen appears to support the maintenance of mass and strength, metabolic function and connective tissue collagen turnover and incorporation (Chidi‐Ogbolu & Baar, 2019). Oestrogen also renders ligaments and tendons more compliant in females compared with males (Hansen & Kjaer, 2016). More compliant connective tissues increase joint laxity, which is likely to increase the risk of anterior cruciate ligament injury (Myer et al., 2008b). Female athletes are more susceptible to knee injuries (Deitch et al., 2006), commonly to the anterior cruciate ligament (Arendt et al., 1999; Matzkin & Garvey, 2019), than age‐matched male athletes. In contrast, males experience greater inflammation after extreme muscle loading (Stupka et al., 2000) and a higher incidence of muscle strains in practice and competition (Cross et al., 2013; Dalton et al., 2015). To complicate matters further, the effect of the menstrual cycle on skeletal muscle stiffness is unclear, with some studies demonstrating varied active and passive whole‐muscle stiffness throughout the menstrual cycle (Ham et al., 2020) and others demonstrating no change (Bell et al., 2011). Oral contraceptive use significantly attenuates cyclical oestrogen fluctuations and has therefore been studied as a possible approach to minimizing soft‐tissue injury risk (Konopka et al., 2019; Morse et al., 2013). However, to date, consensus has not been reached, with some studies demonstrating altered muscle stiffness during oral contraceptive use (Morse et al., 2013) and others reporting no change (Bell et al., 2011). Collectively, these studies suggest that biological sex does impact skeletal muscle stiffness, but the underlying mechanisms are poorly understood. Importantly, whether biological sex mediates an acute reduction of muscle stiffness following fatiguing exercise is also unclear.

Skeletal muscle stiffness is impacted by intracellular (sarcomeric proteins) and extracellular (extracellular matrix) elements, both of which are dynamic. In cases of chronic effectors of stiffness, such as ageing (Noonan et al., 2020b; Pavan et al., 2020; Wood et al., 2014) and physical exercise training (Noonan et al., 2020a), altered stiffness is commonly attributed to the remodelling of extracellular matrix collagen, although it is unclear how this might apply at the single‐fibre level (Noonan et al., 2020b). Interestingly, differences in cellular stiffness have been observed in chemically permeabilized single skeletal muscle fibres across age (Lim et al., 2019; Noonan et al., 2020b) and training status (Noonan et al., 2020*a*), suggesting that intracellular proteins also contribute to chronic stiffness adaptations. Within muscle fibres, passive stiffness is determined primarily by the viscoelastic protein titin (Lim et al., 2019; Ottenheijm et al., 2012). All three titin isoforms [two cardiac (N2B and N2BA) and one skeletal (N2A)] contain extensible (I‐band) and non‐extensible (A‐band and M‐band) regions. Titin isoform distribution can shift as a mechanism for tuning the stiffness of a muscle tissue. In cardiac muscle, shifts occur between N2B and N2BA isoforms (Bupha‐Intr et al., 2011), and in skeletal muscle the N2A isoforms vary in terms of titin splice variants that differ in size (Prado et al., 2005). Acutely, titin extensible regions are subject to post‐translational modifications, many of which have the potential to alter titin‐based stiffness in response to stimuli such as fatiguing exercise (Müller et al., 2014).

Titin‐based mechanisms of altered passive stiffness have been studied extensively in cardiac muscle, owing to the clinical implications of altered cardiac titin for cardiomyopathies (Fukuda et al., 2005; Granzier & Irving, 1995; LeWinter & Granzier, 2014; Müller et al., 2014; Yamasaki et al., 2002). However, the role of titin in modulating skeletal muscle stiffness has been of growing interest, revealing altered titin‐based stiffness in conditions such as cerebral palsy (Mathewson et al., 2014) and Ehlers–Danlos syndrome (Ottenheijm et al., 2012). In both skeletal and cardiac muscle, the most studied titin post‐translational modification is phosphorylation. Phosphorylation sites have been identified in the immunoglobulin (Ig), PEVK and unique sequence regions of the elastic I‐band of titin, but the locations of these phosphorylation sites vary across titin isoforms (Hamdani et al., 2017). One group (Müller et al., 2014) demonstrated that a single bout of exercise was sufficient to modify titin phosphorylation in association with altered myocyte stiffness in murine cardiac muscle. These results suggest that altered titin phosphorylation might contribute to acute, exercise‐induced changes in muscle stiffness. Furthermore, such changes might have implications for whole‐muscle stiffness, given the influence of titin on whole‐muscle mechanical properties (Brynnel et al., 2018).

A growing body of literature supports the notion that titin influences cardiac (Fukuda et al., 2005; Granzier & Irving, 1995; LeWinter & Granzier, 2014; Müller et al., 2014; Yamasaki et al., 2002) and skeletal (Brynnel et al., 2018; Mathewson et al., 2014; Müller et al., 2014; Ottenheijm et al., 2012) muscle stiffness in passive conditions. Our overall approach was to leverage prior investigations suggesting that fatigue, as a physiological stimulus, might alter titin phosphorylation and that modifications to titin might impact tissue stiffness. The contribution of acute titin phosphorylation to regulation of skeletal muscle stiffness in humans remains unclear and, to our knowledge, has never been assessed at the cellular level. Therefore, the purpose of this study was to compare cellular passive stiffness, quantified as the passive Young's modulus to account for potential variation in single‐fibre size, in skeletal muscle samples obtained from post‐fatigue (PF) and non‐fatigued control (C) vastus lateralis muscles of healthy, young males and females. To interrogate potential mechanisms contributing to altered stiffness following fatigue, titin phosphorylation was compared in PF versus C skeletal muscle samples using liquid chromatography coupled with high‐resolution mass spectrometry (LC‐MS). We hypothesized that fatiguing exercise would reduce the passive modulus in single fibres from males and females in conjunction with increased titin phosphorylation.

## 2 MATERIALS AND METHODS

### 2.1 Population

This protocol was approved by the Institutional Review Board at the University of Oregon. Eight young (18–21 years) males (*n* = 4) and females (*n* = 4) from the University of Oregon and surrounding community consented to participate in this study. All participants were recreationally active but reported no participation in structured physical exercise and no resistance training. Thus, they were characterized as ‘untrained’. Self‐reported physical activity was confirmed by ActivePal (Pal Technologies, Glasgow, UK) accelerometers as described previously (Dowd et al., 2012). To limit the potential for menstrual cycle‐dependent variation in circulating oestradiol to contribute to variability in skeletal muscle mechanical properties, all female volunteers self‐reporting eumenorrhoea (*n* = 3) were tested in the prefollicular phase of the menstrual cycle (within 5 days of menses onset). The remaining female volunteer reported use of hormonal contraceptive, and study timing was not considered with respect to menstrual cycle. Participants reported no orthopaedic limitations (severe osteoarthritis, joint replacement or other orthopaedic surgery in the previous 6 months), endocrine disease (hypo/hyperthyroidism, Addison's disease or Cushing's syndrome), uncontrolled hypertension (>140/90 mmHg), neuromuscular disorder, significant heart, liver, kidney or respiratory disease and/or diabetes. Participants were not tobacco smokers and had no current alcohol disorder. Finally, participants taking medications known to affect muscle stiffness or β‐adrenergic signalling of neuromuscular activation (including but not limited to β‐blockers, calcium channel blockers and muscle relaxers) or anabolic steroids were not included.

### 2.2 Study design

Participants visited the laboratory on two occasions separated by ≥1 week. During the first visit, volunteers habituated to measures of voluntary strength, power and the fatigue of their dominant knee extensors (KEs). During the second visit, volunteers performed measures of maximal voluntary isometric KE strength followed by fatiguing exercise to task failure. Fatiguing exercise was followed by bilateral, percutaneous needle muscle biopsies: the first on the exercised limb immediately following fatiguing exercise (PF) and the second on the contralateral, non‐exercised limb (C).

### 2.3 Fatigue protocol

Participants exercised the dominant limb on a Biodex System 3 dynamometer (Biodex Medical Systems, Shirley, NY, USA). Participants were seated on the dynamometer with hips and knee flexed at 90° (180 = full extension). Prior to fatiguing exercise, participants completed three maximum voluntary isometric contractions (MVICs) of the KEs while analog voltage data for torque were sampled at 500 Hz via an analog‐to‐digital converter (Cambridge Electronic Design, UK). Real‐time visual feedback was provided to the participant to encourage maximal effort during the MVIC. The average torque value of the three MVICs was used to set the applied load to 30% MVIC maximal torque for the bout of fatiguing exercise. Following initial MVICs, participants performed repeated, voluntary knee extensions at a rate of one contraction per 1.5 s at this isotonic load until task failure. Task failure was identified as the inability to perform knee extension through ≥50% of the range of motion or to maintain pace with cues to extend their leg on three successive contractions. For seven of the eight participants, fatigue was quantified as the fatigue ratio (final power/initial power), where initial power represents the average peak power of the first five knee extensions performed during fatiguing exercise, and final power represents the average peak power from the last five knee extensions. Owing to an error in data transfer, the signals related to angular velocity and power were not appropriately saved to allow assessment of the fatigue ratio or initial power of one young female. The time to fatigue (task failure) was recorded for all participants.

### 2.4 Muscle biopsy procedure

Percutaneous biopsy of the vastus lateralis muscle was performed in sterile conditions as previously detailed (Tarnopolsky et al., 2011). After sterilization of the biopsy site and injection of local anaesthetic (1% or 2% lidocaine hydrochloride; Hospira Worldwide, Lake Forest, IL, USA), a small (∼5 mm) incision was made in the skin and muscle fascia, allowing passage of a Bergström biopsy needle (5 mm in diameter) to the belly of the vastus lateralis muscle at a depth of ∼2–3 cm. A muscle biopsy of the PF limb was acquired immediately after task failure. The time between the end of exercise and the biopsy was recorded in five of the eight volunteers (three males). Biopsies were recovered between 6 min 30 s and 13 min 40 s (average = 8 min 29 s). Timing was not different between groups (males, 9 min 11 s; females, 7 min 27 s). Thereafter, the C biopsy sample from the contralateral limb was obtained.

### 2.5 Tissue processing

Vastus lateralis biopsy typically yields between 100 and 150 mg (wet weight) of muscle tissue. After samples were removed from the biopsy needle, the tissue was divided for proteomics (∼30 mg), single muscle fibre mechanics (∼40 mg) and electron microscopy (EM; ∼10 mg). The samples intended for proteomics analyses was immediately flash‐frozen in liquid nitrogen and stored at −80°C. The sample intended for mechanical experimentation was placed in dissecting solution (MDS; 120.782 mM sodium methanesulfonate [NaMS], 5.00 mM EGTA, 0.118 mM CaCl_2_, 1.00 mM MgCl_2_, 5.00 mM ATP‐Na_2_H_2_, 0.25 mM KH_2_PO_4_, 20.00 mM BES and 1.789 mM KOH) and parsed into bundles of ∼50 fibres. Bundles were tied to glass rods, chemically skinned overnight, and advanced through solutions of increasing glycerol content before long‐term storage in 50% glycerol solution (5.00 mM EGTA, 2.50 mM MgCl_2_, 2.50 mM ATP‐Na_2_H_2_, 10 mM imidazole, 170.00 mM potassium propionate, 1.00 mM sodium azide and 50% glycerol by volume) at −20°C. The sample apportioned for EM was tied to a glass rod, stretched slightly, and stored at 4°C in Karnovsky's solution until embedding and sectioning as described elsewhere (Miller et al., 2009).

### 2.6 Single‐fibre morphology and contractile measures

Prior to mechanical assays, fibre bundles and dissected single fibres were chemically skinned (MDS + 1% Triton X‐100) before being transferred to plain MDS at ∼4°C until experimentation. Prepared fibres were mounted in relaxing solution (67.286 mM NaMS, 5.00 mM EGTA, 0.118 mM CaCl_2_, 6.867 mM MgCl_2_, 0.25 mM KH_2_PO_4_, 20.00 mM BES, 0.262 mM KOH, 1.00 mM dithiothreitol, 5.392 mM Mg‐ATP, 15.00 mM Creatine Phosphate [CP] and 300 U/mL Creatine Phosphokinase [CPK]) between a force transducer and a length motor (Aurora Scientific, Aurora, ON, Canada) using the Moss clamp technique (Moss, 1979). Multiple wells were present under the mounting surface, allowing rapid transfer of the fibre between relaxing, pre‐activating (81.181 mM NaMS, 5.00 mM EGTA, 0.012 mM CaCl_2_, 6.724 mM MgCl_2_, 5.00 mM KH_2_PO_4_, 20.00 mM BES, 1.00 mM dithiothreitol, 5.397 mM Mg‐ATP, 15.00 mM CP, 300 U/mL CPK) and activating (57.549 mM NaMS, 5.00 mM EGTA, 5.021 mM CaCl_2_, 6.711 mM MgCl_2_, 5.00 mM KH_2_PO_4_, 20.00 mM BES, 9.674 mM KOH, 1.00 mM dithiothreitol, 5.437 mM Mg‐ATP, 15.00 mM CP, 300 U/mL CPK) solutions. The mounting chamber contained a glass bottom and two prisms mounted to the side walls to allow for viewing of the fibre in top‐down and side‐view orientations. Fibre dimensions were measured as follows: *d*_top_ = average of three diameter measures along the fibre using the top‐down view; *d*_side_ = average of three diameter measures along the fibre using the side view; and fibre length = distance between the two trough edges (Callahan et al., 2015). Diameter measures were used for calculation of stress (force per unit area, in kilopascals), and fibre length was used to calculate passive strain (change in length divided by original length, %*L*_0_). All mounted fibres were set to a sarcomere length (SL) of 2.65 µm. This SL is optimal for length–tension generation in human skeletal muscle (Burkholder & Lieber, 2001). Active tension was measured at an SL of 2.65 µm by moving the fibre to pre‐activating solution followed by activating solution until a steady‐state tension was recorded. All fibres were activated (pCa 4.5) prior to passive stretching to measure active tension and confirm fibre viability.

### 2.7 Passive stretch protocol

**FIGURE 1:**
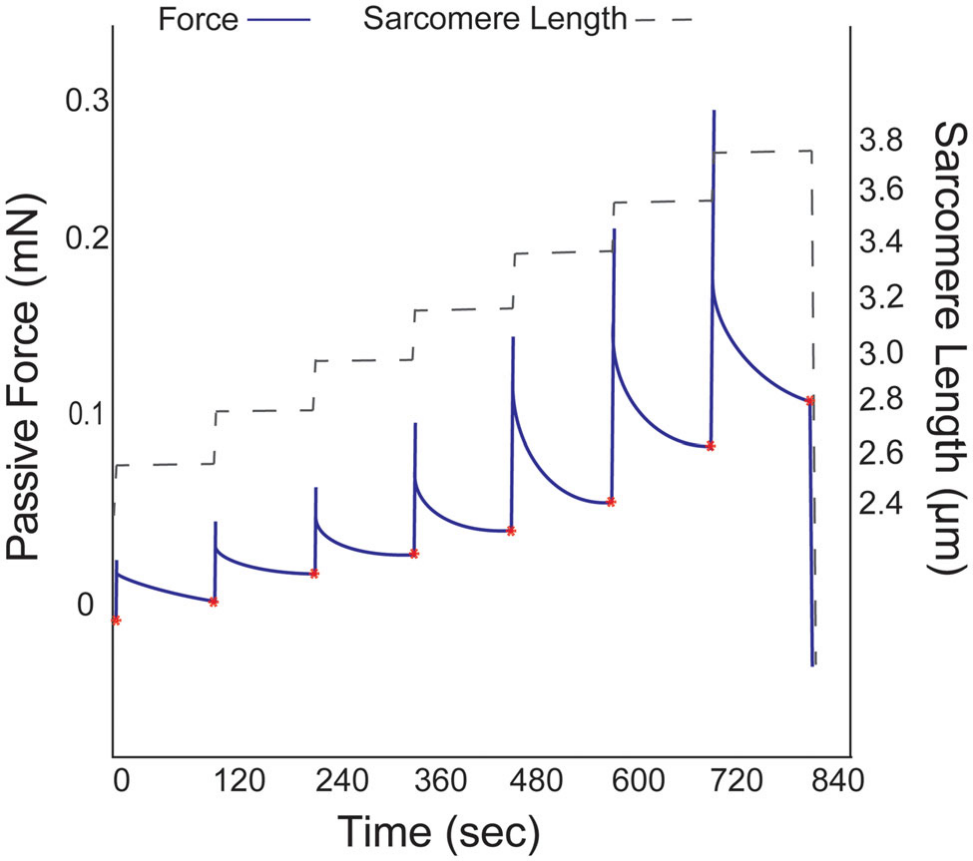
Sample force and sarcomere length traces produced during the passive stretch protocol. The force values measured at the end of each force decay, indicated by red asterisks, were used to calculate the passive stress and, subsequently, passive Young's modulus.

Passive modulus measurements were performed in relaxing solution (pCa 8.0) using a passive stretch protocol adapted from previous work (Lim et al., 2019). The initial sarcomere length was set to 2.4 µm, followed by seven incremental stretches to reach a final length of 156% of initial length (SL ∼3.7 µm). Each stretch lengthened the sample by 8% of initial length and held this position for 2 min of stress‐relaxation (Figure 1). The force response was measured by a force transducer and the change in fibre length was measured by the displacement of the length motor. Sarcomere length was measured throughout the protocol using an inverted microscope located beneath the single‐fibre rig. To determine the extent to which acto‐myosin interactions contributed to measures of passive modulus, a subset of fibres from two males was subjected to the passive stretch protocol in relaxing solution with the addition of 40 mM 2,3‐butanedione monoxime (BDM), a myosin inhibitor. After completion of the passive stretch protocol, each fibre was collected and placed in gel loading buffer (2% SDS, 62.5 mM Tris, 10% glycerol, 0.001% Bromophenol Blue and 5% β‐mercaptoethanol, pH 6.8), centrifuged (3800 *g*, 1 min) and heated at 65°C for 2 min, then stored at −80°C until later assessment of myosin heavy chain (MHC) isoforms. Any fibres failing to demonstrate an increased force in response to fibre stretch (i.e., the subsequent force value after stress‐relaxation was less than that of the previous stretch step) were excluded from analyses. The measured stress at the end of stress‐relaxation was used for subsequent calculations and analyses. Data files were analysed using custom code in Matlab software (R2020b; The MathWorks, Inc., Natick, MA, USA).

### 2.8 Identification of MHC isoforms

**FIGURE 2:**
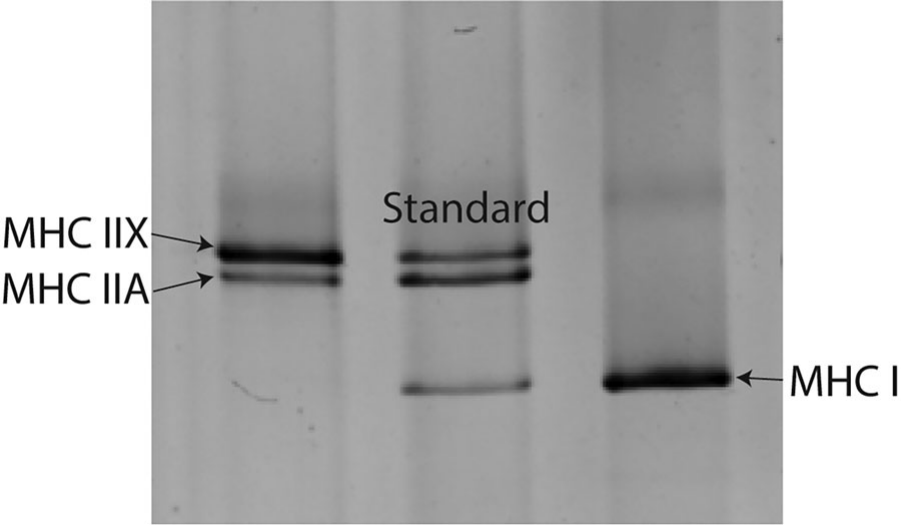
Sample image of silver‐stained myosin heavy chain (MHC) bands. The left lane contains protein from a hybrid single fibre exhibiting both MHC IIA and MHC IIX isoforms. The middle band is a sample homogenate used to visualize all three MHC isoforms (I, IIA and IIX). The right lane contains protein from a single fibre expressing only the MHC I isoform.

SDS‐PAGE was used to assess the MHC isoform of single muscle fibres. A sample from each fibre was loaded into its own well of a 4% stacking/7% resolving polyacrylamide gel. The gel was run at 70 V for 3.5 h followed by 200 V for 20 h at 4°C (Miller et al., 2010). Gels were stained with a silver stain kit (Pierce Biotechnology, Waltham, MA, USA), and the resulting MHC isoform (I, IIA and/or IIX) expression was determined by comparison to a standard made from a multi‐fibre homogenate (Figure 2).

### 2.9 Mass spectrometry assessment of titin phosphorylation

Mass spectrometry was performed on skeletal muscle biopsy samples from individuals who were not included in the single fibre mechanics assays. This separate sample included four young (24.7 ± 5.7 years old), recreationally active individuals (two males and two females) with a body mass index of 24.4 ± 3.4 kg/m^2^. LC‐MS was performed as previously described (Paulo et al., 2015) to determine which titin serine residues were differentially phosphorylated between PF and C samples. Briefly, samples were disrupted by shearing with glass beads, followed by protein digestion via trypsin and phosphopeptide purification by binding to TiO_2_ beads. Phosphopeptides were then labelled with one of 10 different tandem mass tags, combined into a single sample, and run via the Orbitrap Fusion mass spectrometer. Informatics methods (Plubell et al., 2017) were used to determine relative changes in phosphorylation of serine, threonine and tyrosine residues.

### 2.10 Electron microscopy

Electron microscopy was performed in a subset of volunteers (C and PF samples from two female volunteers) to assess the sarcomere ultrastructure (Miller et al., 2009). At the time of biopsy, samples apportioned for EM were fixed in Karnovsky's solution (2.5% glutaraldehyde, 2.5% formaldehyde and 0.1 M sodium cacodylate). Before imaging, samples were treated with 2% osmium tetroxide in 0.5 M sodium cacodylate buffer (Ted Pella), stained with uranyl acetate and embedded in epoxy resin. Cross‐sections (∼100 nm thick) were then cut using an ultramicrotome and contrasted with uranyl acetate before mounting on copper grids and subsequent imaging with a FEI Tecnai with iCorr integrated light and transmission electron microscope. Qualitative assessment of sarcomere ultrastructure was used to check for disruption resulting from fatiguing exercise.

### 2.11 Outcome measures

Passive stress at each SL was calculated as *F*/CSA, where *F* indicates the measured force value at the end of stress relaxation (Figure 1) and CSA indicates fibre elliptical cross‐sectional area. The $\mathrm{CSA}=\pi \times (\frac{d_{\mathrm{top}}}{2}\times \frac{d_{\mathrm{side}}}{2})$, where *d*_top_ is the average of three top diameter measures, and *d*_side_ is the average of three side diameter measures. Strain was calculated as Δ*L*/*L*_0_, where *L*_0_ indicates the initial length. Passive stiffness was quantified as Young's modulus to account for potential differences in fibre size across samples. The passive Young's modulus was calculated as the slope of the stress–strain relationship at short fibre lengths (strain = 1.0%–1.24%*L*_0_) and at long fibre lengths (strain = 1.32%–1.56%*L*_0_). Separate slopes were calculated for short and long fibre lengths to consider the length dependence of cellular passive modulus measures (Noonan et al., 2020b). Additionally, the response of the cellular passive modulus to fatiguing exercise was quantified for each participant by expressing the mean value for PF passive modulus as a percentage of the mean value for C sample modulus. Maximally activated tension was quantified as the measure of steady‐state active force divided by fibre CSA. Titin phosphorylation, assessed via LC‐MS, was quantified as the fold difference between PF and C samples.

### 2.12 Statistical analyses

Statistical testing was conducted using the SPSS software package (SPSS, IBM Corp., Armonk, NY, USA), unless otherwise specified. Anthropometric measures and activity data were compared between males and females using Student's two‐tailed, independent *t*‐tests in Excel. To evaluate differences in the single‐fibre passive modulus at short and long lengths, a linear mixed model was run, with fatigue, biological sex and interaction terms as fixed effects and with participant identity (ID) as a random effect to account for fibre variation within individuals, as described previously (Callahan et al., 2015). Subsequent analyses to investigate an interaction between biological sex and fatigue did so using separate linear mixed models in males and females, each including fatigue as a fixed effect and participant ID as a random effect. To test whether the passive modulus was affected by treatment with BDM in a subset of fibres, a mixed‐effects model was run, with fatigue condition and BDM treatment as main effects and with participant ID as a random effect. To determine whether maximally activated tension, single‐fibre CSA or fibre length differed by biological sex or fatigue condition, separate linear mixed‐effects models were run, with sex, fatigue condition and interaction terms as main effects and with participant ID as a random effect. To assess titin phosphorylation of PF versus C samples using LC‐MS, mean titin phosphorylation was quantified as the fold change ratio, and any significant differences between PF and C samples were identified by a false detection rate (FDR) of <0.05. These LC‐MS statistics were conducted using EdgeR software, proprietary to the Orbitrap Fusion.

## 3 RESULTS

### 3.1 Descriptive measures

The eight participants included were an average of 20.1 ± 1.1 years old (males, 20.8 ± 0.5; females, 19.5 ± 1.3 years; *P* = 0.147). Body mass index (*P *< 0.01), height (*P* = 0.02) and weight (*P *< 0.01) were greater in males versus females (Table 1). Participants were not engaged in structured exercise training, and activity levels of individuals were confirmed by accelerometry. There were no significant differences in step count (*P* = 0.646) and minutes spent in light (<75 steps/min; *P* = 0.137), moderate (75–125 steps/min; *P* = 0.424) or vigorous (>125 steps/min; *P* = 0.445) activity between males and females (Table 1). Despite efforts to select comparable numbers of MHC I and MHC II (including IIA, IIX and A/X) fibres (Privett et al., 2024), MHC I fibres were under‐represented in this sample (10% of total sample), especially in the male participants (3% of fibres from males). However, fibres expressing MHC IIA or MHC IIA/X were represented to a similar extent in samples from males and females. Therefore, only fibres expressing MHC IIA and MHC IIA/X (*n* = 129) were included in statistical analyses (Table 2). There was a trend for reduced single‐fibre CSA in females compared with males (*P* = 0.059), but no difference was observed between fatigue conditions (*P* = 0.566). Fibre length did not differ by biological sex (*P* = 0.168) or fatigue condition (*P* = 0.172).

**TABLE 1:** Anthropometric and activity data of included participants.

Sex	*n*	Body mass index (kg/m^2^)^a^	Height (cm)^a^	Weight (kg)^a^	Step count (steps/day)	Light activity (min/day)	Moderate activity (min/day)	Vigorous activity (min/day)
Male	4	24.4 ± 1.2	185.3 ± 13.0	84.0 ± 10.1	7108 ± 1525	37.6 ± 6.7	50.8 ± 14.7	1.1 ± 1.2
Female	4	21.2 ± 0.3	160.3 ± 5.5	54.4 ± 3.4	7750 ± 2174	26.9 ± 10.5	60.7 ± 18.0	0.5 ± 0.7

*Note*: Values are the means ± SD.

^a^ Significant difference between groups (*P *< 0.05).

**TABLE 2:** Summary statistics of fibres analysed.

Sex	Condition	*n*	Cross‐sectional area (mm^2^)	Length (mm)	Myosin heavy chain	
					IIA (N)	IIA/X (N)
Male	C	35	70.8 ± 20.3	1.7 ± 0.4	21	14
	PF	31	72.9 ± 16.0	1.8 ± 0.5	23	8
Female	C	28	39.6 ± 14.5	1.4 ± 0.5	18	10
	PF	35	45.1 ± 11.6	1.5 ± 0.3	21	14

*Note*: Fibre dimensions and isoform from control (C) and post‐fatigue (PF) fibres. Values are the means ± SD.

### 3.2 Whole‐muscle contractile function and fatigue

**FIGURE 3:**
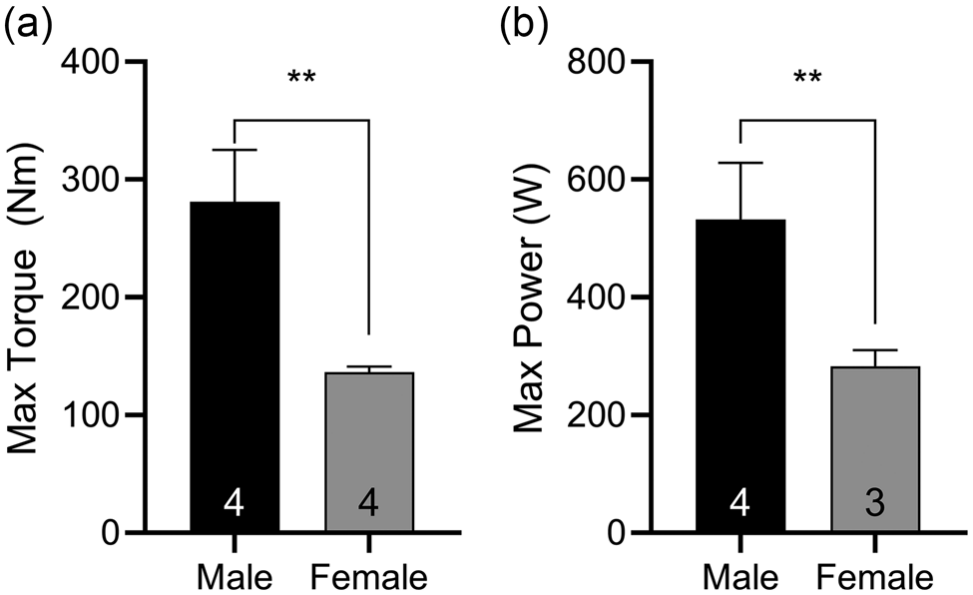
Whole‐muscle function as characterized by maximum voluntary isometric contraction torque and initial power. Males demonstrated significantly greater maximal torque (*P* = 0.001) and maximal power (*P* = 0.008) than females. Data are shown as mean ± SD. ** Indicates significant difference by biological sex (*p* < 0.01).

Knee extensor strength and power were greater in males than females (Figure 3). The average time to fatigue did not differ between males and females (74.6 ± 28.6 vs. 69.4 ± 9.8 s, respectively; *P* = 0.723). There was no difference in the fatigue ratio in the four males and three females for whom data were collected (0.41 ± 0.16 vs. 0.33 ± 0.10 for males and females, respectively; *P* = 0.517). The time between the last knee extension of fatiguing exercise and acquisition of tissue samples was measured in five of the eight volunteers and averaged 8:29 ± 2:57 min, with no difference between males and females (*n* = 3, 9:11 ± 3:55 min and *n* = 2, 7:28 ± 0:47 min, respectively; *P* = 0.599).

### 3.3 Assessment of passive stretch protocol and calculation of Young's modulus

To address potential concerns regarding incomplete stress decay after each applied strain, a third‐order polynomial function was used to perform a non‐linear regression analysis, using the ‘fitnlm’ function in MatLab, on the measured stress decay during the 112 s following applied strain. This produced a predicted final stress value that was compared with the measured stress at the end of each hold period. The difference between the measured stress and predicted stress, termed ‘missed stress’, was calculated at each stretch step, for each fibre. Given the greater stress decay expected at longer lengths (Figure 1), the missed stress values were assessed at strains 1.32%–1.56%*L*_0_. Stress decay was greatest at longer lengths, suggesting the greatest potential for incomplete stress decay. However, measured stress at longer sarcomere lengths differed from predicted stress by 0.6%–2.4%. Given this minimal variation, it is not likely that incomplete stress decay impacted the ability to draw meaningful conclusions from final measured stress, hence measured stress was used throughout. To assess the use of the dual‐slope approach to calculating the passive modulus, the coefficient of determination was calculated for raw data included in the short slope, the long slope and the entire curve (both short and long slopes) for each fibre. The *R* ^2^ values were not obviously different when calculated for the short slope (0.97 ± 0.00), the long slope (0.97 ± 0.01) or the entire curve (0.95 ± 0.00). However, the use of the dual‐slope approach allowed for consideration of the sarcomere length dependence of any significant effects observed (Noonan et al., 2020b). Specifically, the short slope covered the SL range = 2.4 ± 0.03–3.0 ± 0.10 µm and the long slope covered the SL range = 3.2 ± 0.13–3.8 ± 0.18 µm.

### 3.4 Mechanical measures

**FIGURE 4:**
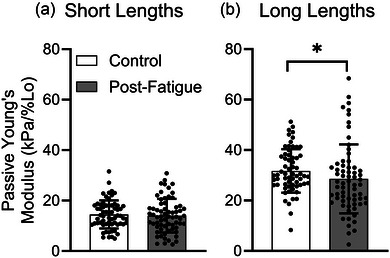
The passive modulus did not differ by fatigue condition at short lengths (a) but was significantly reduced in the fatigued sample at long lengths (b). *Significant difference between fatigued and non‐fatigued single‐fibre passive modulus (*P *< 0.05). Data are shown as the means ± SD.

**FIGURE 5:**
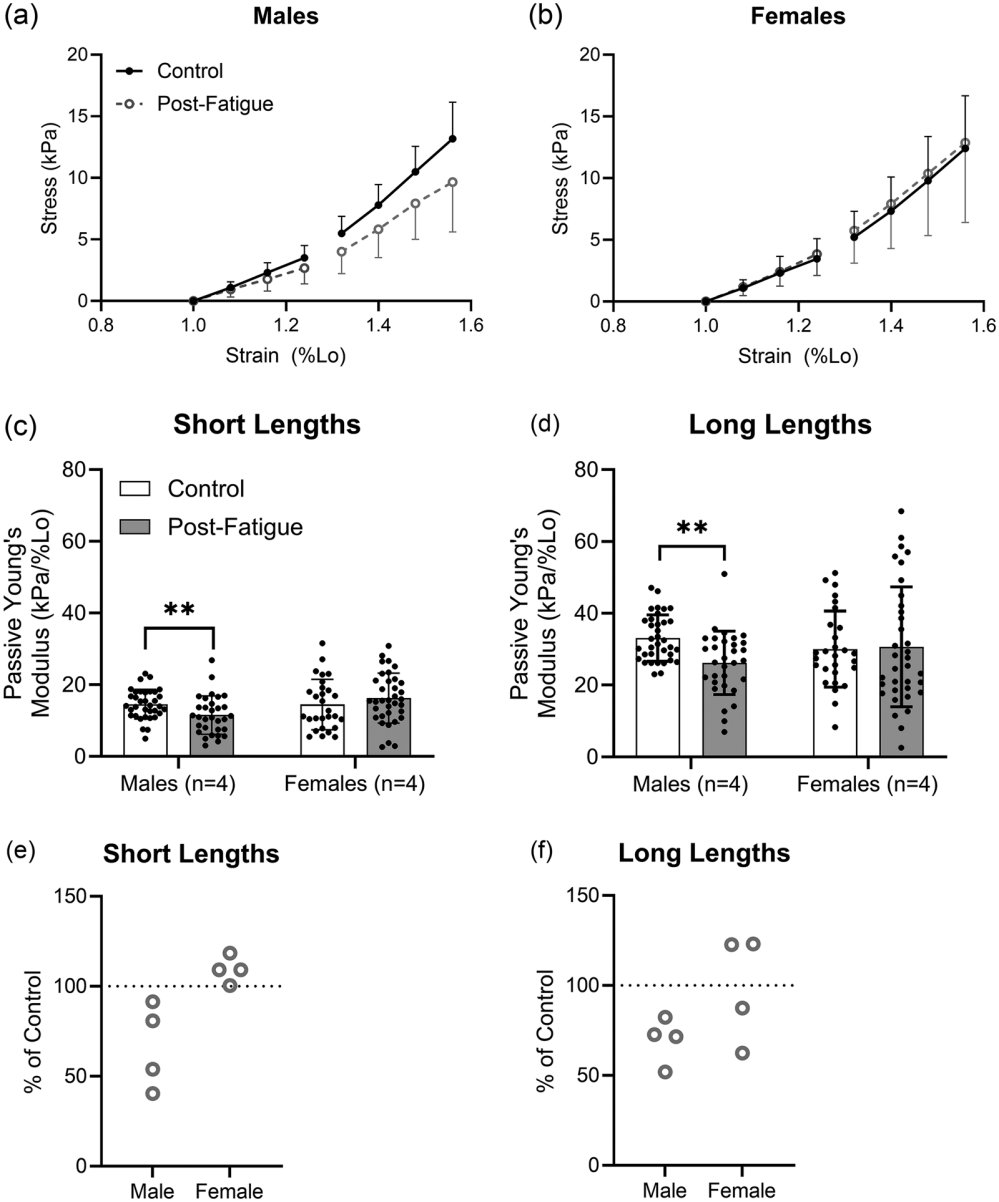
(a, b) Although there was an evident shift in slope of the stress–strain curve between non‐fatigued and fatigued fibres at short and long lengths in the male group (a), there was no such shift in the female group (b). (c, d) Statistical analysis confirmed that fatigue significantly reduced the passive modulus in the single fibres of males at short (c) and long (d) fibre lengths but did not alter the mean modulus in the fibres of females. (e, f) The passive modulus of fatigued fibres was consistently lower compared with non‐fatigued fibres in all four male participants at short (e) and long (f) lengths. However, females exhibited little to no change in the passive modulus at short lengths and variable responses at long lengths. Each individual point in (e, f) represents the average of all fibres for an individual participant. Data are shown as the means ± SD. Asterisks indicate a significant fatigue effect (**P < 0.01).

**FIGURE 6:**
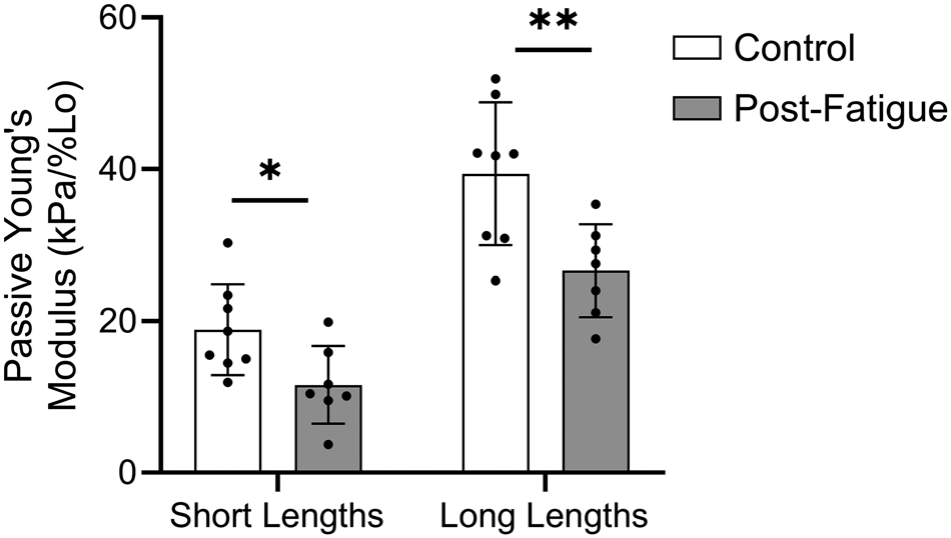
In a subset of fibres that were treated with 2,3‐butanedione monoxime, the passive modulus was significantly reduced in the post‐fatigue (PF) versus control (C) sample at both short and long fibre lengths, suggesting that fatigue‐based differences in the passive modulus persisted regardless of whether myosin was involved. Data are shown as the means ± SD. Asterisks indicate significant differences with fatigue (*P < 0.05 and **P < 0.01).

**FIGURE 7:**
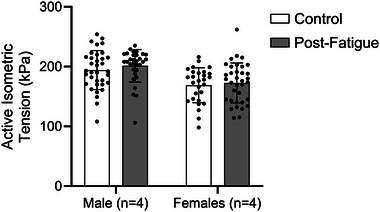
Cellular active tension was not significantly different between males and females (*P* = 0.189) nor between fatigue conditions (*P* = 0.603). Data are shown as the means ± SD.

The MHC isoform was determined by comparison of the band pattern of a single fibre with that of a multi‐fibre homogenate, termed the ‘standard’ (Figure 2), in a silver‐stained acrylamide gel. Because MHC IIA and MHC IIA/X fibres composed the majority of the sample, statistical analyses were conducted on only these fibre types. The passive modulus was not significantly different between males and females at short (*P* = 0.353) or long (*P* = 0.978) fibre lengths. Although the passive modulus was not significantly different in C versus PF fibres at short lengths (C, 14.5 ± 5.6 kPa/%*L*_0_; PF, 14.1 ± 6.7 kPa/%*L*_0_; *P* = 0.341), the passive modulus was significantly reduced in PF fibres at long lengths (C, 31.7 ± 8.6 kPa/%*L*_0_; PF, 28.6 ± 13.7 kPa/%*L*_0_; *P* = 0.043; Figure 4). Because the interaction of fatigue and biological sex was significant at both short (*P* = 0.006) and long (*P* = 0.020) lengths, the effect of fatigue on passive modulus was assessed separately in males and females (Figure 5a, b). Subsequent analyses revealed that fatigue‐induced reductions in passive modulus were driven by males at short lengths (C, 14.6 ± 4.1 kPa/%*L*_0_; PF, 11.6 ± 5.4 kPa/%*L*_0_; *P* = 0.002) and long lengths (C, 33.1 ± 6.5 kPa/%*L*_0_; PF, 26.2 ± 8.8 kPa/%*L*_0_; *P *< 0.001), whereas the modulus in single fibres from females was not significantly different between PF and C at short lengths (C, 14.5 ± 7.0 kPa/%*L*_0_; PF, 16.2 ± 7.0 kPa/%*L*_0_; *P* = 0.263) or long lengths (C, 30.0 ± 10.6 kPa/%*L*_0_; PF, 30.7 ± 16.7 kPa/%*L*_0_; *P* = 0.862; Figure 5c, d). Although males consistently demonstrated a reduced passive cellular modulus in fatigued versus non‐fatigued fibres at short (Figure 5e) and long (Figure 5f) lengths, the response in females varied considerably by individual, especially at long lengths. A separate set of fibres from two of the young males were treated with BDM and subjected to passive stiffness measures. In these samples, the effect of fatigue on cellular passive modulus was maintained at both short (*P* = 0.027) and long (*P* = 0.009) lengths (Figure 6), consistent with observations in the rest of the fibres from the male cohort. In short, BDM did not influence fatigue‐induced reductions in the cellular passive modulus. There was no main effect of biological sex (*P* = 0.189) or fatigue (*P* = 0.603) on active isometric tension in this sample of MHC IIA and MHC IIA/X fibres (Figure 7), suggesting maintained sarcomeric integrity.

### 3.5 Titin phosphorylation

**FIGURE 8:**
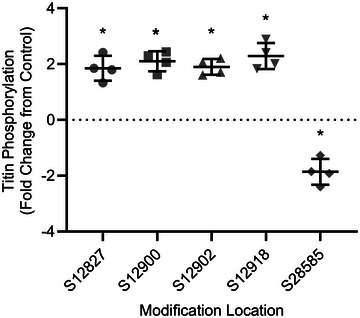
Mass spectrometry analysis demonstrated increased phosphorylation at four serine residues (S12827, S12900, S12902 and S12918) and decreased phosphorylation at serine residue S28525 of the post‐fatigue (PF) versus control (C) sample. At each serine location, every point in the figure represents data from one participant (two males and two females). Bars represent the means ± SD. *Significantly different from C (false discovery rate < 0.05).

Mass spectrometry results (Figure 8) reveal increased phosphorylation of four titin serines [S12827 (FDR = 0.0486), S12900 (FDR = 0.0142), S12902 (FDR = 0.0367) and S12918 (FDR = 0.00195)] and decreased phosphorylation at S28585 (FDR = 0.0367). The Uniprot entry for human titin (entry Q8WZ42; The UniProt Consortium, 2023) suggests that S12827 is within Ig domain 85 and that S12900, S12902 and S12918 are located in between Ig domains 85 and 86. Uniprot also suggests that S28525 is located within Ig domain 132. A previous review summarizing Uniprot data (Hamdani et al., 2017) suggests that in human cardiac titin, S12827, S12900, S12902 and S12918 are located within the elastic I‐band of titin and that S28585 is located within the inelastic A‐band, yet it is unclear how similar serine locations are within titin substructures of cardiac versus skeletal titin isoforms.

### 3.6 Electron microscopy

**FIGURE 9:**
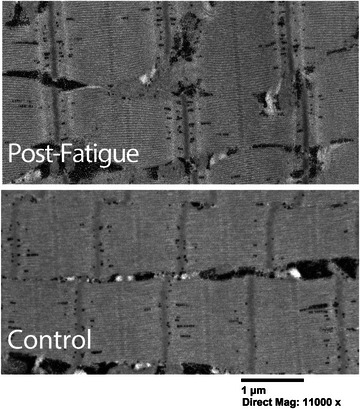
Representative electron microscopy images from fatigued and non‐fatigued skeletal muscle samples did not show evidence of altered sarcomere ultrastructure resulting from the fatigue protocol used in this study.

Electron microscopy images do not demonstrate ultrastructural changes following fatiguing exercise (Figure 9). In contrast to published reports of contraction‐induced damage (Fridén et al., 1983; Fridén, 1984; Roth et al., 1999), which feature disordered sarcomeres and lack of registration between adjacent Z‐lines, the sarcomeres visualized in this study were visibly similar between fatigued and non‐fatigued samples.

## 4 DISCUSSION

### 4.1 Fatiguing exercise induces a reduction in cellular passive modulus

The present results demonstrate a reduction in the passive modulus following fatiguing exercise in permeabilized single muscle fibres from young, untrained adults. Importantly, these observations were made in the absence of exercise‐induced damage to the sarcomere ultrastructure (Figure 9). The observation of comparable active isometric tension in C and PF skeletal muscle fibres (Figure 7) further suggests that fibre integrity was not impaired in our fatigued sample. In these experiments, the use of chemical skinning of dissected single fibres, which permeabilizes the sarcolemma and removes extracellular matrix, supports the idea that reductions in the modulus reflect changes to intracellular proteins, rather than to extracellular matrix mechanical properties. In particular, proteins likely to contribute to altered cellular passive modulus include the contractile proteins actin and myosin and the viscoelastic protein titin.

In a relaxing solution (pCa 8.0), the contribution of residual cross‐bridge formation to cellular passive modulus is possible. This idea was presented initially by Hill (1968) and supported by later work suggesting that a small population of acto‐myosin interactions contributed to force production in relaxed frog skeletal muscle (Campbell & Lakie, 1998). If a proportion of cross‐bridges do remain intact in relaxed muscle fibres, fatigue‐induced post‐translational modifications to myosin, presumably still present at the time of measurement of passive mechanics, might affect changes in the passive modulus via an altered contribution of residual cross‐bridge formation. This possibility of residual cross‐bridge formation was considered in the present study. However, measurement of the passive modulus in relaxing solution with 40 mM BDM, a myosin inhibitor, did not impact the observation of a reduced passive modulus in PF versus C fibres (Figure 6), suggesting that residual cross‐bridge formation was not a primary contributor to fatigue‐induced reduction of the passive modulus. Instead, the reduced cellular passive modulus observed in this sample was more likely to be the result of a non‐contractile intracellular mechanism.

At the cellular level, the passive modulus is determined primarily by the sarcomeric protein titin (Lim et al., 2019; Ottenheijm et al., 2012), and there is evidence to support the role of titin in mediating whole‐muscle passive stiffness (Brynnel et al., 2018). Titin‐based stiffness can be modified by calcium (Nishikawa, 2020), heat shock proteins (Kötter et al., 2014), oxidation (Alegre‐Cebollada et al., 2014; Watanabe et al., 2020) and phosphorylation (Hamdani et al., 2017), all of which are upregulated during fatiguing exercise. Therefore, fatiguing exercise might modify titin‐based stiffness through post‐translational modification of titin elastic domains. For example, exercise increases calcium cycling, prompting calcium‐mediated binding of titin to the thin filament (Dutta et al., 2018) which shortens and stiffens the titin‐based spring during active lengthening of skeletal muscle (Joumaa et al., 2008). Additionally, increased acidity during exercise promotes aggregation of titin Ig domains, which increases titin‐based stiffness (Kötter et al., 2014). However, previous work in human muscle fibres has demonstrated that heat shock proteins also bind to titin Ig domains, thereby stabilizing unfolded domains and preventing aggregation of folded domains (Kötter et al., 2014). In this way, heat shock proteins might prevent the acidification‐based increase of titin‐based stiffness during exercise (Kötter et al., 2014). Exercise also induces oxidative stress, which results in numerous downstream mediators of titin‐based stiffness (Beckendorf & Linke, 2015) including *S*‐glutathionylation, which has been shown to reduce stiffness of muscle cells (Alegre‐Cebollada et al., 2014; Watanabe et al., 2020). In the present study, the storage solutions used included dithiothreitol, an antioxidant, making it unlikely that titin oxidation contributed to the observed changes in the cellular passive modulus. Finally, exercise‐induced β‐adrenergic signalling, coupled with an increased concentration of inorganic phosphate, is likely to contribute to titin phosphorylation (Hamdani et al., 2017; Krüger & Linke, 2006) during fatiguing exercise. Preclinical studies have demonstrated that binding of inorganic phosphate to titin can alter titin‐based stiffness, although the nature of mechanical change is dependent on the location of phosphate binding (Müller et al., 2014). In the present study, five significant phosphorylation changes were identified in the fatigued sample of human skeletal muscle. Therefore, phosphorylation is a possible contributor to the mechanical changes observed after fatiguing exercise.

### 4.2 Fatiguing exercise modifies titin phosphorylation in human skeletal muscle

Mass spectrometry analyses (Figure 8) suggest that the fatiguing exercise undertaken in this study altered titin phosphorylation in human vastus lateralis muscle, and these phosphorylation events might have occurred in the elastic regions of titin. The notion that titin phosphorylation is one of the many post‐translational modifications with the capacity to alter titin‐based stiffness is not new (Hamdani et al., 2017). In fact, it has been proposed previously that phosphorylation of titin Ig domains might reduce the ability of Ig domains to refold (Hamdani et al., 2017), which, given the importance of titin Ig refolding to elastic energy production (Rivas‐Pardo et al., 2016), is likely to reduce titin‐based stiffness. This proposed mechanism is similar to another mechanism described previously (Alegre‐Cebollada et al., 2014; Watanabe et al., 2020), in which titin oxidation via *S*‐glutathione inhibited titin Ig domain refolding and ultimately decreased the stiffness of human cardiomyocytes. Evidence of modified titin‐based stiffness following increased or decreased titin phosphorylation is present in preclinical skeletal (Müller et al., 2014), preclinical cardiac (Fukuda et al., 2005) and human cardiac (Krüger et al., 2009) muscle research, and the specific stiffness response is evidently dependent on the location of the phosphorylation event. In the present study, five serine residues, presumably located within the elastic regions of titin, exhibited increased or decreased phosphorylation in PF versus C skeletal muscle, presenting the possibility that altered titin phosphorylation contributed to the changes observed in the cellular passive modulus. The link between titin phosphorylation and cellular passive mechanics warrants further investigation as a potential mechanism underlying reduced musculoskeletal stiffness after fatiguing exercise.

In the present study, the passive modulus was measured in a subset of single fibres treated with BDM to isolate the specific effects of titin on the passive modulus. Furthermore, fatigue‐induced reductions in the passive modulus were greatest at the longest lengths observed (SL 3.2 ± 0.13–3.8 ± 0.18 µm), where the overlap of thick and thin filaments is minimal and the passive modulus in permeabilized skeletal muscle cells is primarily titin based. Nonetheless, it must be acknowledged that fatiguing exercise has the potential to modify sarcomeric proteins other than titin. Although titin is a likely contributor to the changes in passive mechanical properties observed, future studies in this area must keep in mind that contributions of other proteins are possible.

### 4.3 The effect of fatiguing exercise on the passive modulus is sex specific

The present results suggest that fatigue induces a reduction in skeletal muscle cellular passive modulus in a way that is specific to biological sex (Figure 5a, b). Specifically, the cellular passive modulus was significantly reduced at short (Figure 5c) and long (Figure 5d) lengths in skeletal muscle cells from young, untrained males but not females. The lack of significance might be explained, in part, by the greater variety in response of the passive modulus to fatiguing exercise in females compared with males, especially at longer lengths (Figure 5f). This variation in response might stem from the effects of sex hormones, such as oestrogen, on the skeletal muscle. Given the presence of oestrogen receptors within skeletal muscle and the effects of oestrogen on skeletal muscle strength (Collins et al., 2019), mitochondrial function (Pellegrino et al., 2022; Yoh et al., 2023) and regenerative capacity (Kitajima & Ono, 2016; Pellegrino et al., 2022), oestrogen has a clear effect on skeletal muscle cellular function through modification of intracellular proteins. In fact, oestradiol has been shown to affect regulatory light chain function directly in skeletal muscle (Lai et al., 2016), prompting the possibility that oestrogen might also affect the function of other intracellular proteins, such as titin. In preclinical work using rat cardiac muscle, changes in circulating oestrogen prompted shifts in titin isoform distribution in a way that altered cardiac stiffness (Bupha‐Intr et al., 2011). This result suggests an influential role of oestrogen on the regulation of titin‐based stiffness. However, whether oestrogen directly impacts titin‐based stiffness in human skeletal muscle remains to be seen. Nonetheless, previous work has demonstrated a negative correlation between circulating oestrogen and musculotendinous stiffness (Bell et al., 2012), supporting the notion that oestrogen does impact whole skeletal muscle stiffness. Nonethless, the effect of the menstrual cycle on whole‐muscle stiffness remains unclear, with some evidence of variation in stiffness throughout the menstrual cycle (Ham et al., 2020) and other evidence of no change (Bell et al., 2011). In males, previous work found no relationship between circulating oestrogen and musculotendinous stiffness (Bell et al., 2012). Although the present study design attempted to control for fluctuation in circulating oestrogen throughout the menstrual cycle by collecting biopsies at times of relatively low circulating oestradiol (prefollicular phase or oral contraceptive use), inter‐individual differences in total circulating oestrogen might still have been present in our female participants, perhaps contributing to the diversity in the response of the passive modulus to fatiguing exercise across female participants (Figure 5e and PF). For this reason, future studies of the cellular passive modulus in females should consider measurement of circulating oestrogen at the time of biopsy collection.

### 4.4 Translation

Clearly, measurements made in permeabilized single fibres in passive conditions cannot completely explain the behaviour of whole‐muscle tissue, let alone the in vivo multisystem dynamics that contribute to contractile performance and the risk of soft‐tissue injury. Beyond consideration of anatomical complexity and scale, it must be acknowledged that injuries typically occur during active muscle loading, a condition distinct from our experimental preparation. However, the observed variation in titin behaviour in the PF state might have important implications for active stretch–shortening cycles that impact contractile economy, ballistic movements and injury risk. A growing body of evidence suggests that titin stiffness contributes not only to passive mechanics but also to active contractile function. This is especially evident during active stretch–shortening cycles (Nishikawa, 2020). Indeed, the three‐filament model (Herzog et al., 2012), the winding filament model (Nishikawa et al., 2012) and the dynamic response of titin to cytosolic Ca^2+^ (Dutta et al., 2018; Monroy et al., 2012, 2017) would all suggest that a more compliant titin filament will reduce the capacity for energy storage in the sarcomere and force enhancement following eccentric loading. Additional studies are necessary to test the hypotheses that titin mechanics will impact force‐generating capacity in activating conditions, but the present observations, which are the first of their kind, support the pursuit of these studies. A further consideration when translating our in vitro findings in permeabilized fibres to in vivo function is the time that elapsed between the last fatiguing contraction of the vastus lateralis and acquisition of tissue samples. In this study, that time averaged ∼8.5 min, long enough for significant recovery of the intracellular metabolites that primarily dictate acute muscle fatigue. However, we posit that fatiguing exercise in this context might alter titin phosphorylation in ways that mediate the cellular elastic modulus, and the time course of these alterations is not currently known. The fact that we observed a reduced elastic modulus in fibres from the PF sample, despite this delay in sample acquisition, furthers the notion that the mechanisms dictating protein modification and the elastic modulus are distinct from those dictating acute reductions in myosin cross‐bridge function and subsequent muscle fatigue.

### 4.5 Limitations

A particular strength of the present study is the within‐subject control design. However, broad translation of the present data to the wider population is limited by the relatively small sample from which single fibres were obtained. In addition, our study was conducted on a seemingly uneven distribution of fibre types, potentially limiting our ability to test for a mediating effect of MHC isoform on the response of the cellular passive modulus to fatiguing exercise. Previous work (Miller et al., 2015) suggests that the cellular passive elastic modulus differs among fibre types, highlighting the need to consider fibre type in future studies of the passive modulus. Although the findings are not different when conducted on the complete data set, including fibres of all observed MHC isoforms (I, IIA, IIX, I/IIA and IIA/X), we are, nevertheless, limited in translating observed effects to whole tissue, which is composed of a more even distribution of fibre types. Furthermore, we cannot directly infer tissue‐level data, which includes extracellular matrix, from the behaviour of single cells (Ward et al., 2020), regardless of fibre type. Further studies should interrogate this important factor when considering whether the present data will translate to the whole organism.

In addition, the present study did not find the expected variations in cellular skeletal muscle passive modulus by biological sex. Despite dramatic differences in whole‐muscle contractile function (Figure 3) and single‐fibre size (Table 2), the passive modulus in C was not different by sex. Although variations in fibre swelling when exposed to skinning and relaxing solutions might have complicated interpretation of sex‐based differences at baseline, it could not have explained differences between C and PF fibres, because fibre size was not different between conditions.

Furthermore, our conclusions depend on analysis of distinct, albeit highly similar cohorts. Samples acquired for LC‐MS data were distinct from those used for single‐fibre mechanics, for example. However, the collective findings are consistent with our central hypothesis, and this consistency across separate cohorts can be interpreted as more translatable to a broader population than the same data in fewer volunteers. Collectively, they contribute to a consistent narrative around the acute effects of the PF state in muscle cells, whereby intracellular modification promotes a mechanical phenotype observed in vivo.

Last, it must be acknowledged that although the location and exact sequence of titin serines are based on the highly annotated entry for human titin, Q8WZ42 (The UniProt Consortium, 2023), the precise phosphorylation locations detected in our mass spectrometry analyses, and where they fall along the titin protein, might not yet be fully understood. Specifically, multiple splice variants are likely to be present in the same sample, and shifts in the location of corresponding sequences are likely to complicate our interpretation. Therefore, our ability to speculate regarding potential functional implications of phosphorylation/dephosphorylation at each titin serine is necessarily limited.

## 5 CONCLUSIONS

Collectively, the data presented here suggest a reduction in cellular passive Young's modulus following a single bout of fatiguing exercise in human skeletal muscle. We cannot conclude from the present data that the cellular modulus influences whole‐muscle mechanics, but these observations parallel previous reports of reduced whole‐muscle stiffness following fatiguing exercise (Andonian et al., 2016; Chalchat et al., 2020; Siracusa et al., 2019), supporting the notion that intracellular mechanisms contribute to acute reduction of whole‐muscle stiffness. Furthermore, LC‐MS data suggest that titin phosphorylation is altered at five serine residues during fatiguing exercise, which previous research (Müller et al., 2014) suggests might contribute to altered titin‐based stiffness. Given prior evidence of fatigue‐induced muscle compliance of whole muscle was recorded only in males (Chalchat et al., 2020; Siracusa et al., 2019), it is perhaps not surprising that we observed the effect at the cellular level only in male participants. Perhaps more interesting, the clear link between muscle fatigue and injury risk (Mair et al., 1996) might also be related to sex‐based differences in the dynamic response of muscle fibre compliance to fatigue. Indeed, although the biomechanical aetiology of soft‐tissue injury risk is complicated, sex‐dependent variation in fatigue‐induced compliance provides a tantalizing link to sex‐based variation in soft‐tissue injury. Our study attempted to limit variation in tissue mechanics in female participants by performing our measurements at a time of presumably limited circulating oestradiol. However, circulating oestradiol was not measured directly, and significant variation among and between female participants is possible. Perhaps this variation in circulating oestradiol contributed to the inter‐individual variation in fatigue response in female participants (Figure 5e, f). Future studies of the cellular passive modulus in females should consider whether circulating oestrogen concentrations alter the response of the cellular passive modulus to fatiguing exercise, because this might contribute to the increased incidence of soft‐tissue injury in female athletes compared with their male counterparts (Arendt et al., 1999; Deitch et al., 2006; Matzkin & Garvey, 2019) or the risk of falls and resulting injury in older females (Franse et al., 2017; Stevens, 2005). Last, it remains to be seen whether the observations of an altered passive modulus at the cellular level translate to the tissue level of skeletal muscle, an important step to understanding the interplay between intracellular and extracellular contributors to skeletal muscle stiffness and how they might be altered acutely by fatiguing exercise. Ultimately, the study of mechanisms underlying fatigue‐induced reductions in skeletal muscle passive stiffness will contribute to efforts aimed at reducing fatigue‐induced injury risk in athletes and the risk of falls in older adults.

## AUTHOR CONTRIBUTIONS

These experiments were conducted in the Muscle Cellular Biology Laboratory in the University of Oregon Human Physiology Department. Damien M. Callahan conceived and directed the study. Grace E. Privett, Austin W. Ricci, Karen Wiedenfeld Needham, Larry L. David and Yong How Tan designed and performed experiments. Grace E. Privett, Austin W. Ricci, Karen Wiedenfeld Needham, Larry L. David, Yong How Tan, Karina H. Nakayama and Damien M. Callahan analysed and interpreted study data and revised the manuscript. Grace E. Privett drafted the manuscript. All authors approved the final version of the manuscript and agree to be accountable for all aspects of the work in ensuring that questions related to the accuracy or integrity of any part of the work are appropriately investigated and resolved. All persons designated as authors qualify for authorship, and all those who qualify for authorship are listed.

## CONFLICT OF INTEREST

The authors declare no competing interests.

## Data Availability

The raw data that support the findings of this study are available from the corresponding author upon reasonable request.
